# Phonon-assisted upconversion in twisted two-dimensional semiconductors

**DOI:** 10.1038/s41377-022-01051-9

**Published:** 2023-01-02

**Authors:** Yuchen Dai, Pengfei Qi, Guangyi Tao, Guangjie Yao, Beibei Shi, Zhixin Liu, Zhengchang Liu, Xiao He, Pu Peng, Zhibo Dang, Liheng Zheng, Tianhao Zhang, Yongji Gong, Yan Guan, Kaihui Liu, Zheyu Fang

**Affiliations:** 1 School of Physics, State Key Laboratory for Mesoscopic Physics, Academy for Advanced Interdisciplinary Studies, Collaborative Innovation Center of Quantum Matter, Nano-optoelectronics Frontier Center of Ministry of Education, Peking University, 100871 Beijing, China; 2 Institute of Modern Optics, Nankai University, Tianjin Key Laboratory of Micro-scale Optical Information Science and Technology, 300350 Tianjin, China; 3 Photonics Research Center, School of Physics, MOE Key Lab of Weak-Light Nonlinear Photonics, and Tianjin Key Lab of Photonics Materials and Technology for Information Science, Nankai University, 300071 Tianjin, China; 4 School of Materials Science and Engineering, Beihang University, 100191 Beijing, China; 5 Center for Physicochemical Analysis and Measurements in ICCAS, Analytical Instrumentation Center, Peking University, 100871 Beijing, China

**Keywords:** Nanophotonics and plasmonics, Nonlinear optics

## Abstract

Phonon-assisted photon upconversion (UPC) is an anti-Stokes process in which incident photons achieve higher energy emission by absorbing phonons. This letter studies phonon-assisted UPC in twisted 2D semiconductors, in which an inverted contrast between UPC and conventional photoluminescence (PL) of WSe_2_ twisted bilayer is emergent. A 4-fold UPC enhancement is achieved in 5.5° twisted bilayer while PL weakens by half. Reduced interlayer exciton conversion efficiency driven by lattice relaxation, along with enhanced pump efficiency resulting from spectral redshift, lead to the rotation-angle-dependent UPC enhancement. The counterintuitive phenomenon provides a novel insight into a unique way that twisted angle affects UPC and light-matter interactions in 2D semiconductors. Furthermore, the UPC enhancement platform with various superimposable means offers an effective method for lighting bilayers and expanding the application prospect of 2D stacked van der Waals devices.

## Abstract (web summary)

Rotation-angle-dependent phonon-assisted photon upconversion in twisted two-dimensional semiconductor is investigated. Lattice relaxation along with enhanced pump efficiency, lead to an inverted contrast between upconversion and conventional photoluminescence.

## Graphical abstract

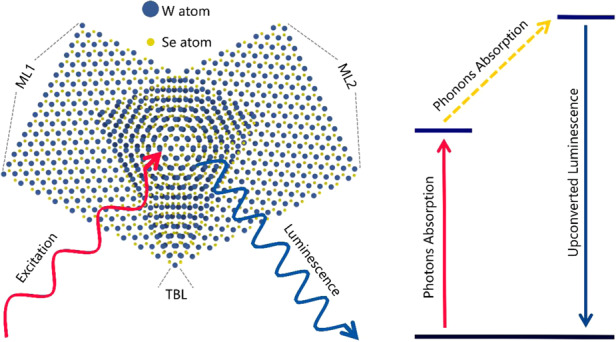

## Introduction

**Fig. 1:**
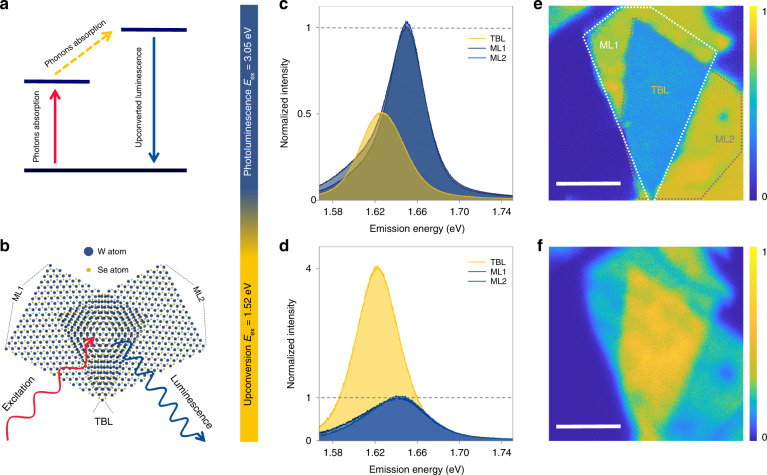
Phonon-assisted upconversion enhancement in a 5.5° twisted bilayer WSe_2_. **a** Schematic of phonon-assisted upconversion mechanism. **b** Schematic of the device structure. **c**, **d** PL (**c**) and UPC (**d**) spectra of ML1(dark blue lines), ML2 (blue lines) and TBL (yellow lines), gray dotted lines indicate intensities normalized as 1. **e**, **f** PL (**e**) and UPC (**f**) maps of the corresponding bilayer sample in **c** and **d**, scale bar 5 μm

Photon upconversion (UPC) is a light-matter interaction process in which photons emit at an energy higher than incident photons. The phenomenon has been widely observed in materials such as Organic dyes^1^, Carbon nanotubes^2^, Quantum dots^3^, II-VI semiconductors^4^, Rare-earth-doped materials^5^, and Two-dimensional materials^6^, presenting a broad application prospect in Energy harvest^1^, Biosensing^7^, Display^8^, Laser^9^, and Optical manipulation^10^. Origins of UPC can be attributed to many mechanisms including triplet-triplet annihilation^11^, high-order harmonic generation^12^, multiphoton absorption^13^, Auger recombination^14^, and phonon-assisted anti-Stokes emission^15^. Among them, phonon-assisted UPC absorbs one or more phonons from lattice thermal vibration to achieve energy gain of light emission, has been regarded as a promising way to realize optical microzone refrigeration (Fig. 1a).

Recently, single- and multi-phonon-assisted UPC in transition metal dichalcogenide (TMD) monolayers have attracted intensive interest^16,17^. Compared with conventional semiconductors, TMD monolayers possess stronger optical transition strength and phonon-exciton interaction^18^, thereby providing an ideal platform with enhanced light-matter interaction^19^. Besides, Owing to reduced dielectric screening and enhanced quantum confinement, strong Coulomb attraction leads to excitons with enormous binding energy in TMD monolayers, opening opportunities for exploring exciton physics and devices at room temperature^20^. Furthermore, Weak van der Waals interactions between TMD layers encourage optionally stacking with other 2D materials^21,22^, enabling interlayer rotation angle to be a new degree of freedom for tailoring optoelectronic properties. Novel physical phenomena including moiré excitons^23–25^, moiré polaritons^26^, shear solitons^27^, and correlated states^28^, have been observed in twisted angle TMDs. However, whether phonon-assisted UPC is affected by interlayer rotation angle of 2D semiconductors remains unclear.

In this work, we study phonon-assisted UPC in WSe_2_ twisted bilayers, an inverted contrast between phonon-assisted UPC and conventional photoluminescence (PL) emergent is observed at room temperature. A 4-fold UPC enhancement is achieved in a 5.5° twisted bilayer while PL weakened by half, the UPC intensity ratio of bilayer/monolayer is 8-fold higher than that of PL. Here, we attribute the phenomenon to a mutual influence of both UPC pump efficiency increasing caused by spectral redshift and interlayer exciton conversion efficiency decreasing resulting from lattice relaxation in WSe_2_ twisted bilayers. As a new phenomenon in twisted-angle TMDs, the results provide a novel insight into a unique way that twisted angle affects UPC and light-matter interactions in 2D semiconductors. Simultaneously, the counterintuitive phenomenon provides an effective method for light up bilayer TMDs to expand application prospects of 2D stacked van der Waals devices including versatile quantum light source^22,29^, artificial excitonic crystals^23^, and high-performance semiconductor lasers^30^. In addition, sufficient phonon-assisted UPC enhancement directly observed in large areas of exposed material can easily superimpose with other enhancement techniques such as optical cavities^31^, plasmon resonance^32^, quantum dots^33^ for an even more appealing enhancement.

## Results

### Phonon-assisted upconversion enhancement in WSe_2_ twisted bilayer

The twisted WSe_2_ homogenous structures were constructed by using dry transfer method (see method section). All WSe_2_ monolayers used were mechanically exfoliated from single-crystal bulks to guarantee sample quality. By deliberately stacking, each sample retains three regions for characterization: the lower monolayer (ML1), upper monolayer (ML2), and twisted bilayer (TBL) (Fig. 1b). The interlayer rotation angles were characterized and controlled by adopting the hexagonal symmetric polarization second harmonic generation (SHG) patterns^34^ of WSe_2_ (Fig. S2).

The spectra of WSe_2_ twisted bilayer reveal the inverted contrast between PL and UPC. The PL (UPC) measurements used a femtosecond pulse with ~3.05 eV (~1.52 eV) as excitation light. Figure 1c, d depicts PL and UPC spectra from a WSe_2_ twisted bilayer with a 5.5 ° rotation angle, respectively. Blue (yellow) solid lines represent the spectra from two MLs (TBL). Quantitatively, UPC intensity in TBL demonstrates a 4-fold enhancement than that in ML, and the TBL/ML intensity ratio of UPC is 8 times higher than that of PL. Corresponding PL and UPC maps (Fig. 1e, f) revealed more clearly the significant enhancement of UPC and the inverted contrast between PL and UPC. Besides, the qualities of WSe_2_ monolayers used to construct homogenous structures were tightly controlled, spectra and maps show that the PL and UPC intensities of two MLs are very close enough to eliminate the possibility of a fake enhancement caused by differences in monolayer qualities. In addition, UPC enhancement in TBL cannot be attributed to the intensity superposition of two uncombined individual monolayers since the intensity of UPC is 2-fold higher than that of the case, and simultaneous enhancement of PL and UPC was not observed.

It is counterintuitive that UPC and PL show no simultaneous enhancement or weakening effects in the absence of resonant condition, and luminescence in bilayer is stronger than in monolayer TMD homogenous structures. Here, we attribute this phenomenon to the mutual influence of both the increase of UPC pump efficiency caused by spectral redshift and the decrease of interlayer exciton conversion efficiency resulting from lattice relaxation.

The multi-phonon-assisted upconversion observed in both WSe_2_ MLs and TBLs originates directly from the simultaneous absorption of photon and phonons by valence electron near K-valley^35^. The initial states of upconversion cannot be intermediate states resonating with excitation photon, since excitation energy is set at 100+ meV below neutral excitons, and resonance at such a high energy gain has not been reported. It has been observed that electron-electron scattering can contribute to upconversion at lower temperatures with moderate concentrations of resident electrons. The photogenerated electrons with enough energy can achieve the energy gain of neutral exciton through twice spin-conserving valley switchings with resident electrons along with the formation of dark spin-forbidden intravalley exciton and dark momentum forbidden intervalley exciton^36^.

### Pump efficiency and phonon participation in UPC

**Fig. 2:**
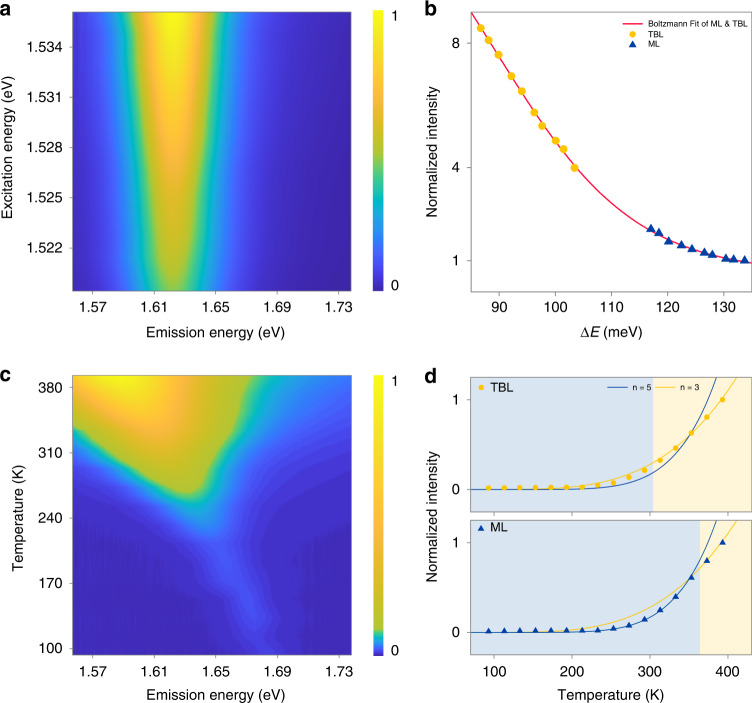
Energy gain and temperature dependence of UPC in a 5.5° twisted bilayer WSe_2_. **a** Excitation-energy-dependent UPC spectra in the WSe_2_ TBL. **b** Energy-gain-dependent UPC intensity plots of TBL (yellow dots) and ML (blue triangles), the intensity at the highest energy gain of ML is normalized as 1, the red solid line fits both ML and TBL results as a whole based on the Boltzmann model (Table S1). **c** Temperature-dependent UPC spectra in the WSe_2_ TBL. **d** Temperature-dependent UPC intensity plots of TBL (top panel) and ML (bottom panel); the blue (yellow) solid line represents the fitting based on Bose-Einstein distribution when the number of participating phonons *n* = 5 (*n* = 3)

The intensity of phonon-assisted photon upconversion is significantly affected by pump efficiency in the excitation process. The spectra redshift of 31 meV in TBL (Fig. 1d), which is mainly due to the increased content of low-energy trions^37^ (see Fig. S3) originates from the more multifarious configurations formed^38^, reduces the required energy gain between emission and excitation photons, resulting in pump efficiency improving and the enhancement of UPC in TBL region. Figure 2a depicts excitation energy-dependent UPC spectra of a 5.5° TBL (see Fig. S4 for ML). In the range of excitation (1519 meV to 1536 meV), peak energy and width of UPC spectra remain unaffected while the intensity enhanced obviously as excitation energy gradually increases and approaches to emission photon. For clarity, Fig. 2b shows the relationship between UPC integral intensity and energy gain ΔE between emission and excitation peaks, where yellow dots (blue triangles) represent the results of TBL (ML), solid red line is fitting based on the Boltzmann model. Within the excitation energy blueshift range of 17 meV, UPC intensities of both ML and TBL nearly doubled, which suggests that the effect of energy gain is significant. Conventional PL is not as sensitive to excitation energy as UPC, therefore PL intensity remains basically unchanged when UPC is significantly enhanced due to emission photon redshift, leading to the inverted contrast between UPC and PL in TBL and MLs.

The microcosmic mechanism of UPC enhancement caused by spectral redshift originates from the reduced phonon number involved in upconversion assistance. Both ML and TBL show considerable UPC energy gain of more than 100 meV at room temperature (Fig. 1d), while A1 phonon with the highest intensity in WSe_2_ is only 30 meV, so there must be multiple phonons absorbed simultaneously. The temperature-dependent UPC spectra for 5.5° TBL were measured and depicted in Fig. 2c (see Fig. S4 for ML) to reveal the effects of energy and density of phonons. With temperature increase, UPC peak position apparently redshifted, and spectra are broadened, which attribute to temperature-related lattice dilatation and electron-lattice interaction in direct bandgap semiconductors. The well-known Varshni equation fits UPC spectra by considering interactions between excitons and longitudinal-acoustical (LA) and longitudinal-optical (LO) phonons^39,40^ (Fig. S5), which is also embodied in PL. It is also noted that due to sharp reduction of phonon density at low temperature and increase of energy gain caused by excitonic spectra blueshift, phonon-assisted UPC intensity dramatically weakens with temperature decreases, which is the unique feature that distinguishes phonon-assisted UPC from PL and two-photon excitation-induced emission. Figure 2d depicts the temperature-dependent UPC intensity of TBL (top) and ML (bottom), respectively, revealing the participation of multiple phonons. The average population of phonon gas in WSe_2_ is proportional to the Bose-Einstein distribution powered by the number of phonons *n*, and solid blue lines (solid yellow lines) in Fig. 2d represent the circumstances *n* = 5 (*n* = 3), respectively. For both ML and TBL, fewer phonons participate in the UPC process as temperature rises, consistent with energy gain decreases and phonons energy increases that lead to UPC enhancement. Most importantly, TBL enters the three-phonon region earlier than ML, suggesting that UPC enhancement in TBL attributed to the decrease of energy gain caused by spectral redshift, originates from the reduced phonon number involved in upconversion assistance.

The reduction of required phonon number caused by spectral redshift in TBL region has been proven to be effective for enhanced UPC, however, it remains insufficient considering that in natural WSe_2_ bilayers, while spectral redshift also exists, both UPC and PL significantly weakened compared with monolayer (Fig. S6). Therefore, it is necessary to consider the role of interlayer rotation angle in physical scenarios.

### Interlayer exciton modulated UPC& PL in WSe_2_ TBLs

**Fig. 3:**
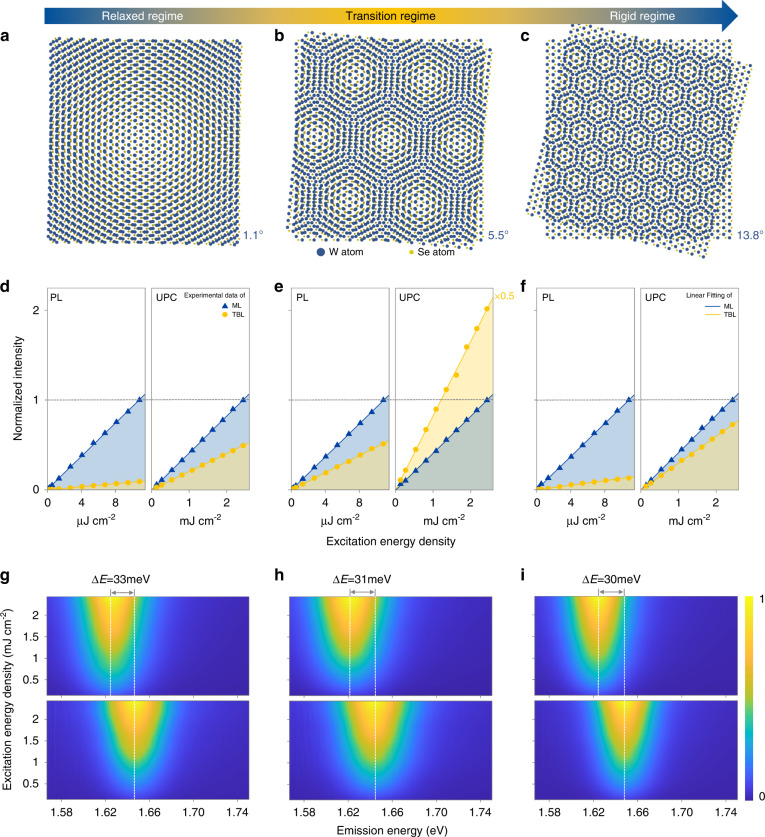
Twist-angle-dependent UPC& PL in WSe_2_ TBLs. **a**–**c** Schematic of WSe_2_ twisted bilayer superlattices with rotation angles of 1.1° (**a**), 5.5° (**b**), and 13.8° (**c**). **d**–**f** Excitation-energy-density-dependent PL (left panels) and UPC (right panels) intensities plots of the 1.1° (**a**), 5.5° (**b**), 13.8° (**c**) WSe_2_ TBLs (yellow dots), and corresponding MLs (blue triangles); the intensities of MLs at the highest excitation power are normalized as 1; the yellow and blue solid lines represent corresponding linear fitting. g-i Excitation-energy-density-dependent UPC spectra of the 1.1° (**g**), 5.5° (**h**), 13.8° (**i**) WSe_2_ TBLs (top panel), and corresponding MLs (bottom panel)

Photon UPC in WSe_2_ bilayers is strongly dependent on interlayer rotation angle. Figure 3 depicts characterizations of representative samples with rotation angles of 1.1°, 5.5°, and 13.8° relative to AB (BA) stacking from left to right, respectively. Fig. 3d–f are variations of PL (left panel) and UPC (right panel) intensity with the excitation energy density of the three samples at room temperature. Blue triangles (yellow dots) represent results in MLs (TBLs). Plots are normalized according to intensities at the highest excitation power in MLs (as shown by gray dotted lines) for clarity. PL and UPC intensities of all samples show a good linear relationship with excitation energy density to eliminate excitonic saturation and nonlinear effects, and it is further confirmed that the UPC here is attributed to multi-phonon assistance^41^ rather than multiphoton absorption or Auger scattering. Notably, only TBL at a rotation angle of 5.5° demonstrated enhanced UPC than ML, while PL intensity predictably weakened in all TBLs. UPC enhancement in TBLs has been repeatedly observed in other samples close to rotation angle of 5.5° (Fig. S7). Figure 3g–i is the corresponding energy-density-dependent UPC spectra of TBL (top) and ML (bottom), indicating that thermal and renormalization effects can be reasonably neglected since excitonic peak position and spectral shape of UPC remain unchanged with excitation power varies. Moreover, UPC spectra of all TBLs redshift about 30 meV relative to corresponding MLs, which explains the enhanced TBL/ML ratio of UPC compared to PL in Fig. 3d–f. However, similar redshifts rule out the possibility that variations of UPC enhancement with rotation angle are due to differences in pump efficiency, indicating that other factors, such as interlayer excitons and lattice relaxation, must be considered.

Intensities of UPC and PL in twisted bilayer WSe_2_ are modulated by interlayer exciton conversion efficiency. The weakening of PL and UPC in natural WSe_2_ bilayers is the result of conversion from direct intralayer excitons to indirect interlayer excitons, then dissipating through non-radiative processes^42^. The enhanced TBL/ML intensity ratio of PL and UPC at a specific rotation angle indicates a significant decrease in conversion efficiency towards interlayer excitons. Quantifying according to UPC, compared with WSe_2_ bilayer with 5.5° rotation angle, the forward interlayer exciton conversion efficiency of 1.1° bilayer is 8-fold higher (5.5-fold according to PL), and that of 13.8° is 5.6-fold higher (3.9-fold according to PL).

Lattice relaxation associated with interlayer twisting of WSe_2_ bilayer is essential for the rotation angle dependence of interlayer exciton conversion efficiency. It has been reported that lattice relaxation of non-rigid bilayer two-dimensional materials during interlayer twisting is especially significant in TMDs^43–45^, which is embodied in the changes of domain shape and domain wall width between AA and AB (BA) stacking, as well as the appearance of microzone strain. The state of TMDs homogeneous structures with rotation angles less than 30° was divided into three regimes: the relaxation, transition, and rigid regime at a small, medium, and high angle, respectively (Fig. 3) in a previous study^45^. Lattice microzone strain increases gradually with rotation angle in relaxation regime, decreases in the rigid regime, and reaches the peak magnitude in the transition regime. Significant microzone strain leads to lattice deformation and damages interlayer flatness, resulting in considerable interlayer separation. The concentration of UPC enhancement in the transition regime (~5.5°) originates from the fact that considerable interlayer separation weakens the bonding of interlayer excitons and reduces their forward conversion efficiency.

### Evolution of lattice relaxation in WSe_2_ TBLs

**Fig. 4:**
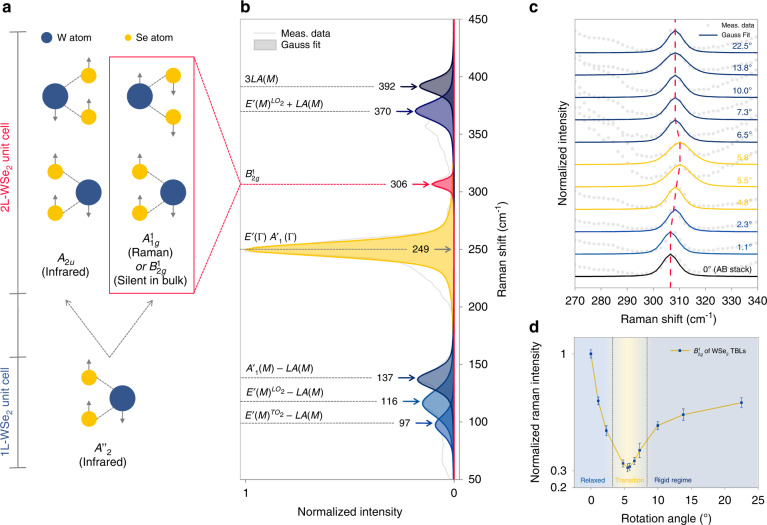
Interlayer rotation angle dependence of Raman spectroscopy in WSe_2_ TBLs. **a** The origin of B^1^_2g_ mode in the 2H-WSe_2_ bilayer. **b** The normalized spectrum of a WSe_2_ bilayer with Raman shifts in the range of 50 to 450 cm^−1^; the identifiable Raman modes are highlighted by Gaussian fitting, and the corresponding symmetry assignments are indicated. **c** The normalized spectra with Raman shifts in the range of 270 to 340 cm^−1^ for WSe_2_ TBLs with a series of interlayer rotation angles; the B^1^_2g_ modes are highlighted by Gaussian fitting; the red dotted line is the visual guide of the B^1^_2g_ Raman shifts evolution. **d** The twist-angle-dependent B^1^_2g_ Raman intensity plot

Raman spectroscopy has been demonstrated to play a vital role in studying 2D twisted systems^45,46^, was used to provide evidence for lattice relaxation associated with interlayer twisting. Figure 4b depicts the spectrum of a bilayer WSe_2_ in a Raman shift range of 50 to 450 cm^−1^, in which a strong Raman peak in the vicinity of 249 cm^−1^, typically generated by E^1^_2g_ and A^1^_g_ modes in WSe_2_, can be clearly observed, as well as a series of combination modes (identifiable symmetry assignments are indicated in Fig. 4b according to literatures^47,48^), illustrating high-quality samples are exfoliated. In addition, bilayer B^1^_2g_ mode near 306 cm^−1^ is observed (Fig. 4b), which split from the A^“2^ mode that comes from inverted out-of-plane vibrations of metal and chalcogenide atoms in monolayer WSe_2_ (Fig. 4a). The B^1^_2g_ mode is invisible to both infrared and Raman in bulks, however, becomes visible with a finite layer number and enhanced with layer number decreases, reaches the strongest in the bilayer, and disappears in monolayer^49,50^. The strong contrast of B^1^_2g_ mode in monolayer and bilayer is essential to reveal lattice distortion and interlayer separation associated with lattice relaxation.

Raman shift and intensity of B^1^_2g_ mode are obviously dependent on rotation angles of WSe_2_ bilayers. Figure 4c depicts normalized rotation-angle-dependent Raman spectra in the range of 270 to 340 cm^−1^, B^1^_2g_ peak positions are emphasized by Gauss fittings, and samples in relaxed (blue), transition (yellow), and rigid regime (dark blue) are marked with different colors. B^1^_2g_ mode shifts up from 306 cm^−1^ in 0 ° AB (BA) stacking, reaches the highest 310 cm^−1^ in transition regime due to lattice distortion resulting from high strain, then decreases in the rigid regime remains near 308 cm^−1^, overall trend is consistent with the variation of microzone strain. Figure 4d depicts the dependence of B^1^_2g_ intensity on rotation angle, intensities are normalized according to the stable E^1^_2g_ mode for rigour. B^1^_2g_ mode intensities show an apparent trend consistent with the variation of Raman shifts, reaching the lowest value (~30% that of AB (BA) stacking) in transition regime and then rebounding. Strong interlayer separation in transition regime weakens the B^1^_2g_ mode intensity evidently due to its Raman invisibility in monolayer. In addition, although the fact that 0 ° sample possesses the highest B^1^_2g_ intensity suggests that the reduction of AB (BA) stacking area proportion also affect B^1^_2g_ mode intensity, the affection is not particularly significant due to the limited rotation angle range. Overall, the rotation-angle-dependent evolution of B^1^_2g_ mode in TBLs provides evidence for the introduction of lattice distortion and interlayer separation associated with lattice relaxation, which reasonably interprets the mechanism of rotation angle dependence of interlayer exciton conversion efficiency in TBLs.

### Interlayer-neutral exciton ultrafast transition

**Fig. 5:**
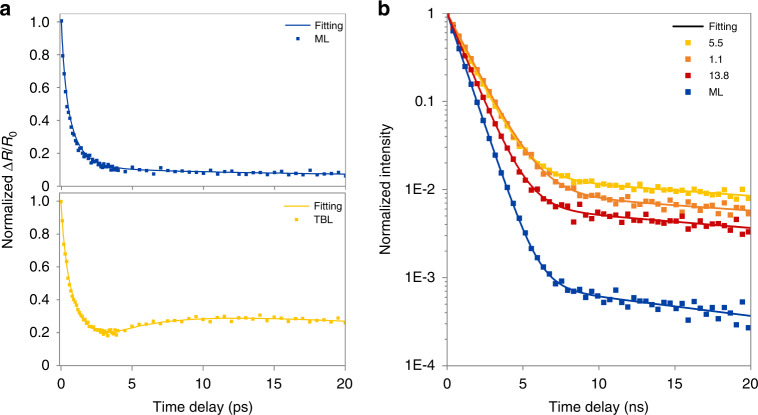
Time-resolved study of WSe_2_ TBLs luminescence. **a** The transient reflected absorption pump-probe detection results of WSe_2_ ML (top panel) and 5.5° TBL (bottom panel) within 20 ps; solid lines represent fittings based on Eq. (1) (Table S1). **b** The fluorescence lifetime measurements of ML (blue) and TBLs (warm colors) with various interlayer rotation angles; solid lines represent biexponential fittings (Table S1)

The weakening of forward conversion from neutral excitons to interlayer excitons at a specific rotation angle by lattice relaxation induced interlayer separation lies at the core of rotation-angle-dependent UPC enhancement, which can be probed by transient absorption pump-probe detection technique. Figure 5a depicts the pre-20ps transient absorption results of ML (top) and 5.5° TBL (bottom) at room temperature, ΔR/R_0_ is the differential reflection intensity of probe beam demonstrating the variation of carrier populations directly (see Methods for more details). The Δ R/R_0_ signal of 5.5° TBL rises again after a sharp decline compared with ML, indicating the conversion of weakly bonded interlayer excitons to neutral excitons leads to a hysteretic recovery of ΔR/R_0_ signal. Excitons in WSe_2_ relaxed from excited electrons and holes either recombine to emit photons via radiation or non-radiatively annihilated via multi-body scattering. Considering non-radiative processes including multi-exciton annihilation and exciton-exciton annihilation^51,52^, along with the formation and decomposition of interlayer excitons, the excitonic kinetic relaxation equation can be written as:

$$\frac{{\partial {{{\mathrm{N}}}}_{x_0}}}{{\partial t}} = - {{{\mathrm{A}}}}N_{x_0}^4 - {{{\mathrm{B}}}}N_{x_0}^2 - \left( {F + {{{\mathrm{{\Gamma}}}}}} \right)N_{x_0} + RN_{Ix}$$

1 $$\frac{{\partial {{{\mathrm{N}}}}_{Ix}}}{{\partial t}} = {{{\mathrm{F}}}}N_{x_0} - \left( {{{{\mathrm{R}}}} + {{{\mathrm{{\Gamma}}}}}^\prime } \right)N_{Ix}$$

where *N*_x0_ and *N*_Ix_ are densities of neutral excitons *x*_0_ and interlayer excitons Ix, *A* is the rate of four excitons annihilation, *B* is that of exciton–exciton annihilation, *F*(*R*) is forward (backward) conversion rate from neutral excitons *x*_0_ to interlayer excitons Ix, Γ(Γ^’^) is recombination rate of neutral excitons *x*_0_ (interlayer excitons Ix). The last term in the equation can be neglected in pre-20ps window since the radiation lifetime of excitons is much longer than other processes. RK method was applied to solve the ordinary differential equation numerically and results fit well with transient pump-probe measurements (Fig. 5a). Weakening of interlayer excitons forward conversion due to interlayer separation makes the backward conversion from interlayer excitons to neutral excitons visible, which is proved by transient absorption detection, provides further evidence for the angle-dependent UPC enhancement mechanism.

Fluorescence lifetime measurements are added as complements to radiation processes of transient absorption pump-probe detections in a long-time window (Fig. 5b), solid lines represent corresponding biexponential fittings. The prolonged fluorescence lifetime curves of TBLs relative to ML are mainly attributed to the high content of long-lived trions in the WSe_2_ bilayer. Although the Morie potential at a low rotation angle may constrain excitons to prolong their lifetime^23^, the effect is insignificant in WSe_2_ TBLs according to corresponding lifetime curves.

## Discussion

This letter studied the phonon-assisted photon upconversion in WSe_2_ twisted bilayers modulated by lattice relaxation and pump efficiency. We summarize physical scenarios of the inverted contrast between UPC & PL: The significant lattice relaxation in WSe_2_ homogeneous structures drives microzone strain to peak at a certain rotation angle. Lattice distortions and interlayer non-flatness associated with peak-strain rotation angle regime bring about considerable interlayer separations. The bonding of interlayer excitons is weakened due to interlayer separation, which reduces the conversion efficiency of direct neutral excitons to indirect interlayer excitons, blocks the non-radiative relaxation of excitons, leads to simultaneous enhancements of UPC and PL. UPC enhanced more obviously due to strong pump efficiency dependence. Simultaneously, increased content of low-energy trions in WSe_2_ bilayer leads to a redshift of excitonic peak to excitation energy, which reduces energy gain. Pump efficiency is significantly enhanced due to the reduction of required assisted phonons, resulting in UPC enhancement, while PL remains unaffected because of weak dependence on pump efficiency. With the mutual influence two processes, the inverted contrast between phonon-assisted UPC and conventional PL emergent in WSe_2_ twisted bilayer of high-strain rotation angle regime. A 4-fold UPC enhancement is achieved in 5.5° twisted bilayer while PL weakened by half. The results provide a novel insight into a unique way that twisted angle affects UPC and light-matter interactions in 2D semiconductors. Furthermore, the UPC enhancement platform with various superimposable means offers an effective method for lighting bilayers and expanding the application prospect of 2D stacked van der Waals devices.

## Materials and methods

### Sample preparation

All samples were prepared by PDMS-assisted dry transfer method. Firstly, WSe_2_ monolayers and hBN few-layers were mechanically exfoliated on PDMS films by using scotch tapes. The monolayer characteristics of WSe_2_ were confirmed by PL spectra. Moreover, thicknesses of hBN few-layers were controlled according to the contrast to eliminate influences of hBN thickness. Next, a precise transfer platform was applied to transfer materials layer by layer to a silicon wafer with 100 nm SiO_2_ on the surface to form the structure of hBN-WSe_2_-WSe_2_-hBN-SiO_2_-Si (Fig. S1). All the above operations were carried out in air at room temperature. Finally, samples were annealed in a high vacuum environment of 200 degrees Celsius for more than 8 hours. The WSe_2_–WSe_2_ rotation angle was measured according to SHG patterns (Fig. S2).

### Micro-PL&UPC spectra measurements

The excitation laser of micro-UPC spectrum measurements was generated by a mode-locked oscillator (Tsunami 3941C-25XP) with tunable wavelength, and the wavelength range used for measurement was 808–816 nm. The excitation laser (407 nm) of micro-PL spectrum measurements was the frequency-doubled beam by a BBO crystal. Excitation lights were focused by an infinity-corrected long work distance micro-objective (Mitutoyo, 100x, NA = 0.5) to excite the sample. An EMCCD camera (Andor Ixon 888) with a micro-objective and matched widefield tube lens (Thorlabs TTL200-A) was used to observe the sample and confirm the excitation position. The PL (UPC) signal was collected by a spectrometer (Acton SP2500) equipped with a liquid-nitrogen-cooled CCD after passing through a 450 nm long-pass edge filter (800 nm short-pass edge filter). The sample was placed in a heating and freezing microscope stage system (LINKAM THMS600), with a temperature range of 80–970 K, with an accuracy of 0.1 K.

### Confocal PL&UPC maps and fluorescence lifetime measurements

PL and UPC maps were measured by an ISS Q2 confocal laser scanning system. Among them, excitation wavelength of PL (UPC) maps was 405 nm (810 nm), and the signal is collected by a Nikon TE2000 microscope with a 60 × NA = 1.2 WI objective lens after passing through a 480 nm long-pass edge filter (810 nm short-pass edge filter). The fluorescence lifetime measurements were collected by the FastFLIM system. The excitation source is a pulsed laser with wavelength 405 nm and frequency 40 MHz. The PL signals pass through a 480 nm long-pass edge filter and are finally collected by Nikon TE2000 microscope with a 60 × NA = 1.2 WI objective lens.

### SHG and Raman Measurements

SHG measurements were realized by a confocal system with a polarization controllable laser. The continuous-wave laser with a wavelength of 1064 nm was incident on the sample through a polarizer and a half-wave plate. The SHG signal generated by the sample was collected after passing through a polarizer parallel to the polarization direction of incident light. The rotation of incident light polarization direction was realized by rotating the half-wave plate. Raman measurement was carried out using a confocal micro-Raman spectrometer (Horiba XploRA Plus). The excitation light was a continuous-wave laser with a wavelength of 532 nm. Raman signals were collected by an objective lens (Olympus MPlan N) with a magnification of 100X, NA = 0.9. The grating constant used was 1800 mm^−1^. All Raman measurements were carried out in air at room temperature.

### Transient reflected absorption pump-probe measurements

The laser generated by a femtosecond pulse laser oscillator (Tsunami 3941C-25XP) (816 nm, 73 fs, 80 MHz) is first divided into a pump beam and a probe beam by a splitter. Among them, the pump beam passes through a BBO crystal to produce a final pump beam with a wavelength of 408 nm. The probe beam is focused into a photonic crystal fiber (Newport SCG-800) to produce supercontinuous white light, and then the final 750 nm probe beam is obtained through a 750 ± 10 nm bandpass filter (Thorlabs FB750-10). The time delay between pump and probe pulse is controlled by a steeper linear stage (Newport M-ILS150PP) on the path of pump beam. In order to filter out the excitation light and the PL, a 1500 Hz chopper is installed on the path of pump beam, and the lock-in amplifier (Stanford SR830) automatically filters out the 1500 Hz signals. The spot size of the probe beam on the sample surface was focused <1 μm. In order to improve signal-to-noise ratio, reflected detection pulse signal passes through a 450 nm long-pass edge filter (Thorlabs FEL0450) and is finally collected by a high-sensitivity photomultiplier tube (Thorlabs PMM02) connected to the lock-in amplifier. Detailed light paths and measurement steps have been reported in the previous work^53^.

## Supplementary information

Supplementary Information
