# Supplementary material for: Phonon-assisted upconversion in twisted two-dimensional semiconductors
Source: Light Sci Appl. 2023 Jan 2;12:6. doi: 10.1038/s41377-022-01051-9 (PMC9806105; doi:10.1038/s41377-022-01051-9)
Supplement: Supplementary file 1 — Supplementary Information [file 41377_2022_1051_MOESM1_ESM.docx]

**Supplementary Information for**

**Phonon-assisted upconversion in twisted two-dimensional semiconductors**

Yuchen Dai,^1^ Pengfei Qi,^1,2^ Guangyi Tao,^1,3^ Guangjie Yao,^1^ Beibei Shi,^1^ Zhixin Liu,^1^ Zhengchang Liu,^1^ Xiao He,^1^ Pu Peng,^1^ Zhibo Dang,^1^ Liheng Zheng,^1^ Tianhao Zhang,^3^ Yongji Gong,^4^ Yan Guan,^5^ Kaihui Liu,^1^ Zheyu Fang^1^*

**^1^ School of Physics, State Key Laboratory for Mesoscopic Physics, Academy for Advanced Interdisciplinary Studies, Collaborative Innovation Center of Quantum Matter, Nano-optoelectronics Frontier Center of Ministry of Education, Peking University, Beijing 100871, China**

**^2^ Institute of Modern Optics, Nankai University, Tianjin Key Laboratory of Micro-scale Optical Information Science and Technology, Tianjin 300350, China**

**^3^ Photonics Research Center, School of Physics, MOE Key Lab of Weak-Light Nonlinear Photonics, and Tianjin Key Lab of Photonics Materials and Technology for Information Science, Nankai University, Tianjin 300071, China**

**^4^ School of Materials Science and Engineering, Beihang University, Beijing 100191, China**

**^5^ Center for Physicochemical Analysis and Measurements in ICCAS, Analytical Instrumentation Center, Peking University, Beijing 100871, China**

* Correspondence to: Zheyu Fang

Email: zhyfang@pku.edu.cn

CONTENTS

[S1. Optical microscopy. 1](#_Toc120021066)

[S2. Interlayer rotation angle characterization via SHG. 2](#_Toc120021067)

[S3. Study on the content of trion X- in the 5.5 ° TBL. 3](#_Toc120021068)

[S4. Excitation energy and temperature dependent UPC of monolayer WSe_2_. 4](#_Toc120021069)

[S5. Evolution of UPC spectra with temperature. 5](#_Toc120021070)

[S6. Scenario in the natural bilayer (AB stacking) WSe_2_. 7](#_Toc120021071)

[S7. UPC enhancements in other transition-regime twisted bilayer samples. 8](#_Toc120021072)

[S8. Summary of fitting parameters. 9](#_Toc120021073)

[References 10](#_Toc120021074)

1. Optical microscopy.


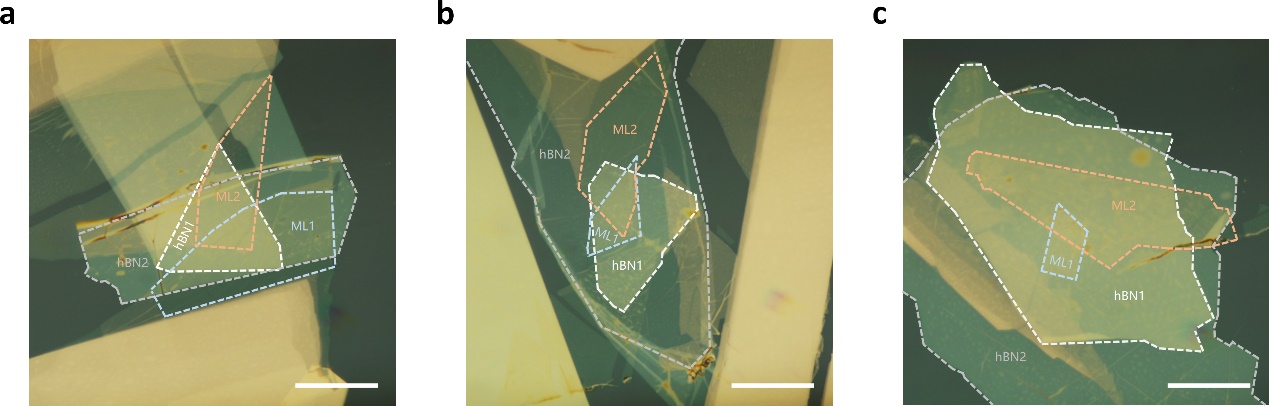


**Figure S1**: Optical microscopy photographs of the representative WSe_2_ TBLs with rotation angles of 1.1° (**a**), 5.5° (**b**), and 13.8° (**c**), scale bar 20 μm.

1. Interlayer rotation angle characterization via SHG.


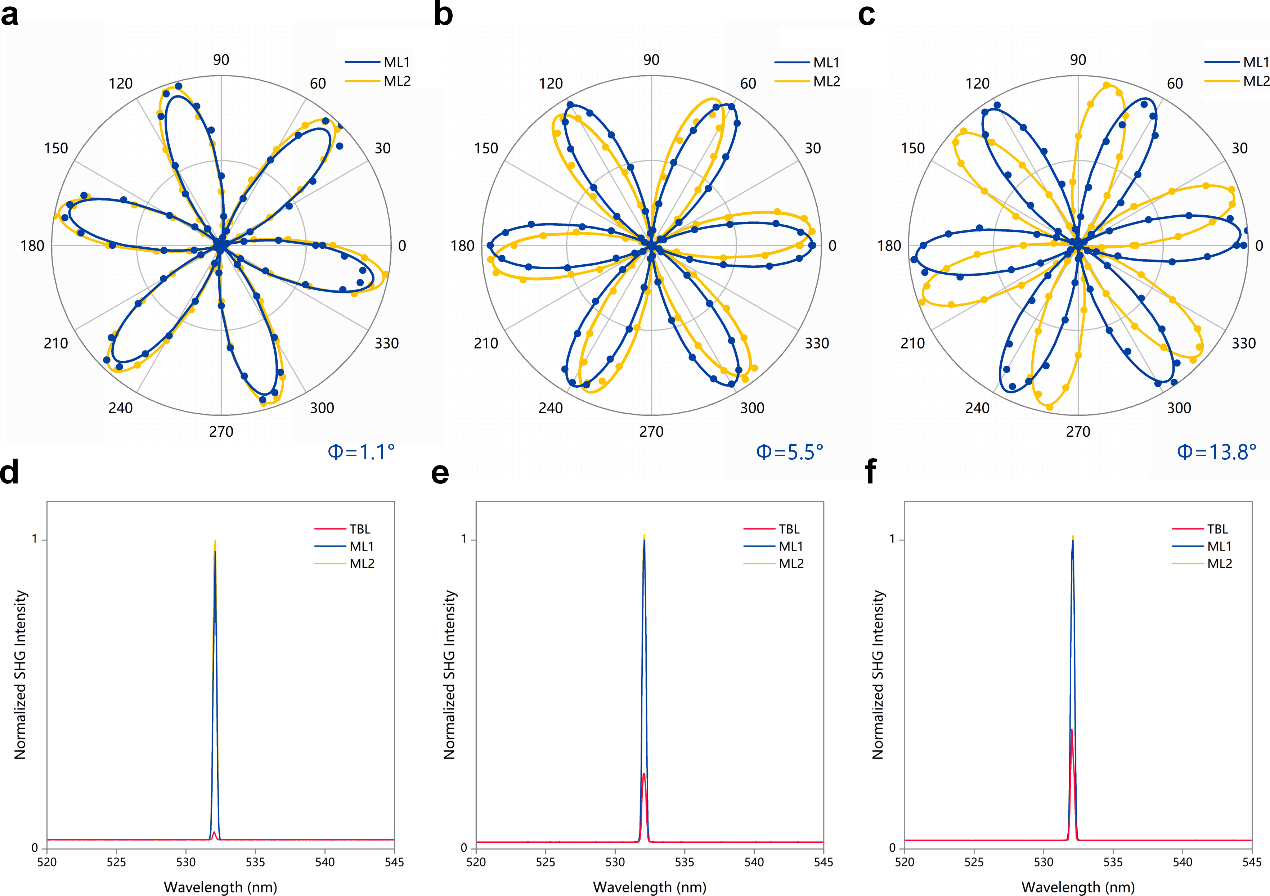


**Figure S2**: **a**-**c**, SHG pattern of the 1.1° (**a**), 5.5° (**b**), and 13.8° (**c**) twisted bilayer samples; the blue and yellow dots represent results of different monolayers in the same sample, respectively; the solid lines are corresponding sinusoidal fittings. **d**-**f**, SHG spectra of pattern peaks from each layer of three samples, the weakening of SHG signals in TBLs reveal that the interlayer rotation angles are near 0°.

1. Study on the content of trion X- in the 5.5 ° TBL.


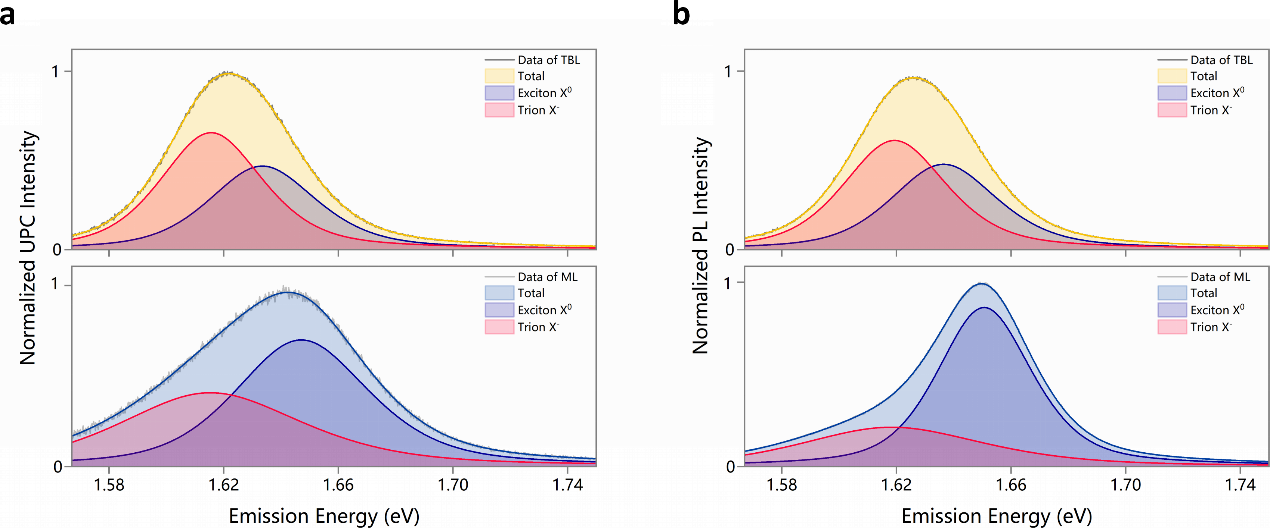


**Figure S3**: Normalized PL (**a**) and UPC (**b**) spectra of WSe_2_ 5.5° TBL (top panel) and ML (bottom panel); the fitting spectra of trion X- (neutral exciton X_0_) are represented by red (dark blue) solid lines.

1. Excitation energy and temperature dependent UPC of monolayer WSe_2_.


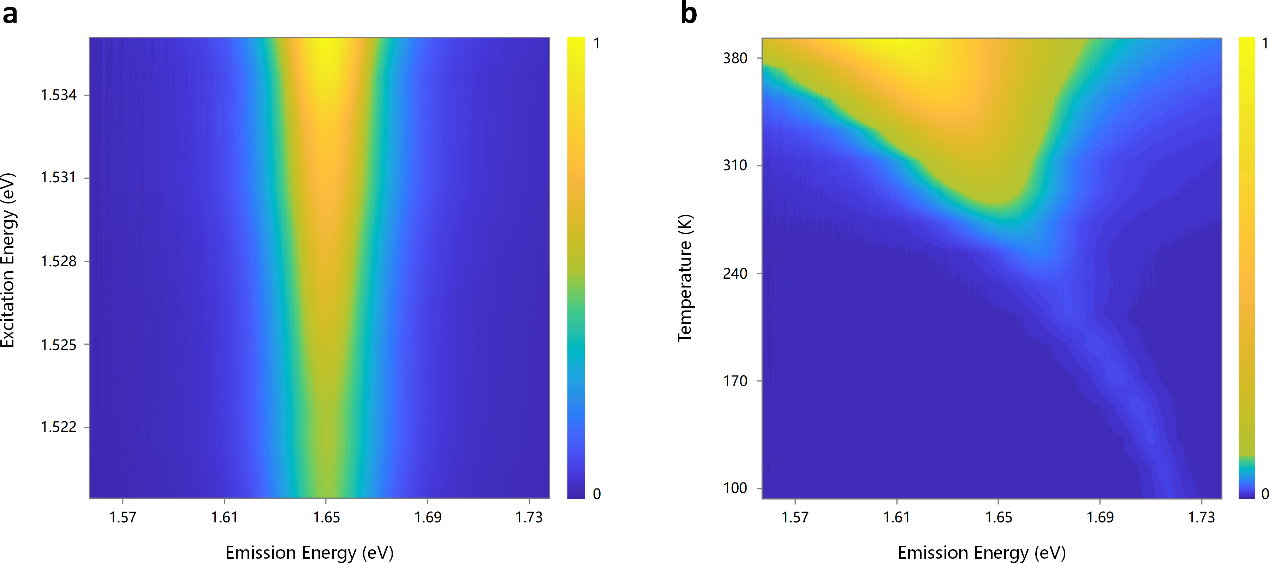


**Figure S4**: Contour Maps of excitation-energy-dependent (**a**) and temperature-dependent (**b**) UPC spectra in the WSe_2_ monolayer.

1. Evolution of UPC spectra with temperature.


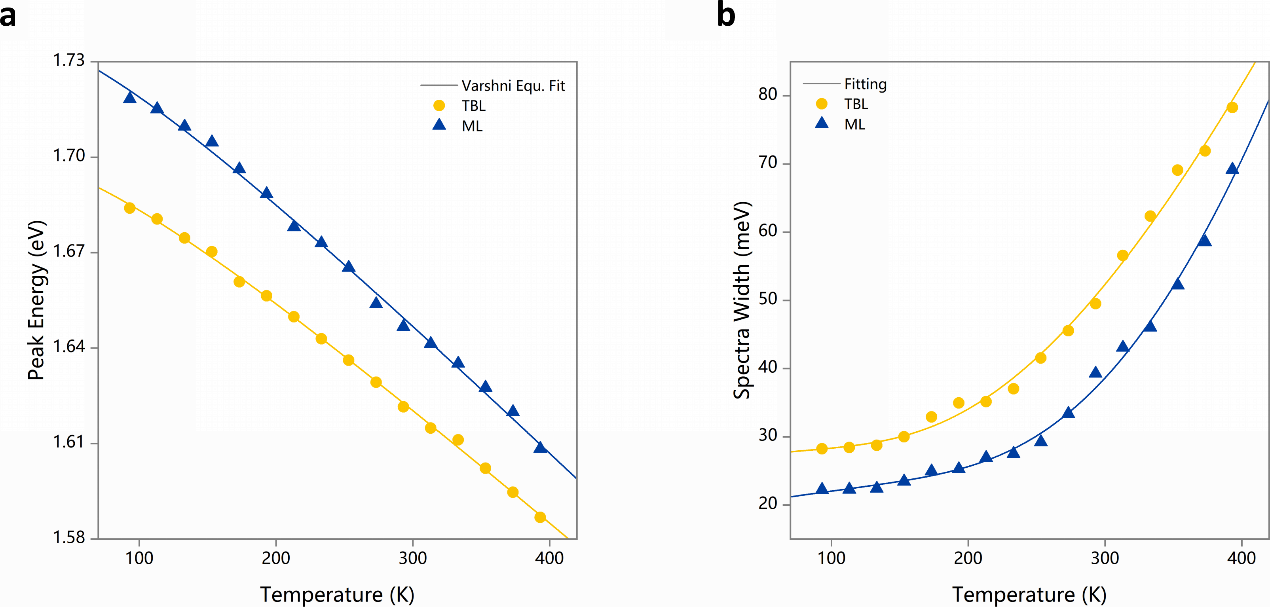


**Figure S5**: The temperature-dependent UPC peak energy (**a**) and spectra width (**b**) plots of 5.5° TBL (yellow dots) and ML (blue triangles); the solid lines represent the corresponding fittings.

The evolution of excitonic peak energy is attributed to temperature-dependent lattice dilatation and electron-phonon interaction, which can be described by the Varshni equation:

$E_{g}\left( T \right)=E_{0}\left( 0 \right)-\frac{\alpha T^{2}}{T+\beta}$ (S1)

Varshni equation describes the temperature-dependent energy gap for semiconductors[^1^](#_ENREF_1). The global optimal parameters of fittings are E_g_ (0) = 1.698 eV, α = 3.8×10^－4^ eV·K^-1^ and β = 135 K for 5.5° TBL (yellow solid line in Figure S5a), and E_g_ (0) = 1.738 eV, α = 4.3×10^－4^ eV·K^-1^ and β = 120 K for ML (blue solid line in Figure S5a).
The broadening of excitonic spectra width is ttributed to the interaction of excitons with the longitudinal-acoustical (LA) and longitudinal-optical (LO) phonons. Accordingly, in semiconductors without considerable impurity doping and defects, the temperature-dependent linewidth of excitons can be described as[^2^](#_ENREF_2):

$\Gamma\left( T \right)=\Gamma_{0}+\gamma_{LA}T+\gamma_{LO}N_{LO}(T)$ (S2)

where the second (third) term describes the contribution of the interaction between excitons and LA (LO) phonons, N_LO_ (T) represents the Bose-Einstein distribution of LO phonons occupation, and the constant term arises from intrinsic imperfections scattering. The corresponding fitting results are Γ_0_ = 26.6 meV, γ_LA_ = 0.02 meV·K^-1^, and γ_LO_ = 4.74 meV for 5.5° TBL (yellow solid line in Figure S5b), and Γ_0_ = 19.3 meV, γ_LA_ = 0.03 meV·K^-1^, and γ_LO_ = 18.18 meV for ML (blue solid line in Figure S5b).

1. Scenario in the natural bilayer (AB stacking) WSe_2_.


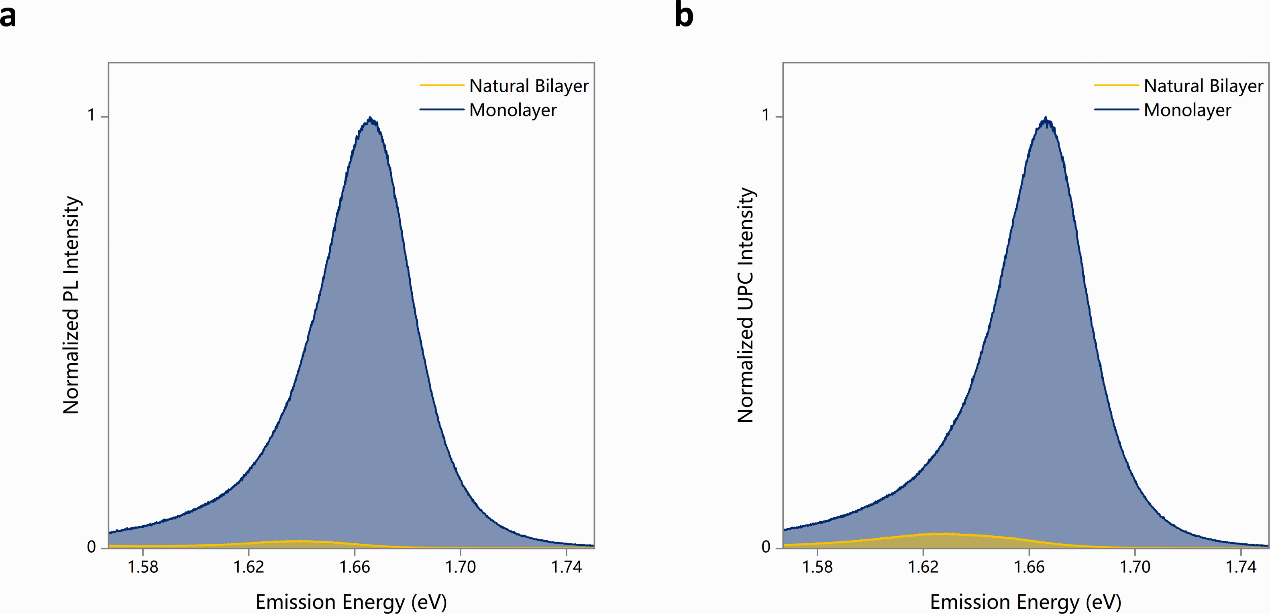


**Figure S6**: PL (**a**) and UPC (**b**) spectra in a natural bilayer (solid yellow lines) and corresponding monolayer (solid blue lines) of WSe_2_.

1. UPC enhancements in other transition-regime twisted bilayer samples.


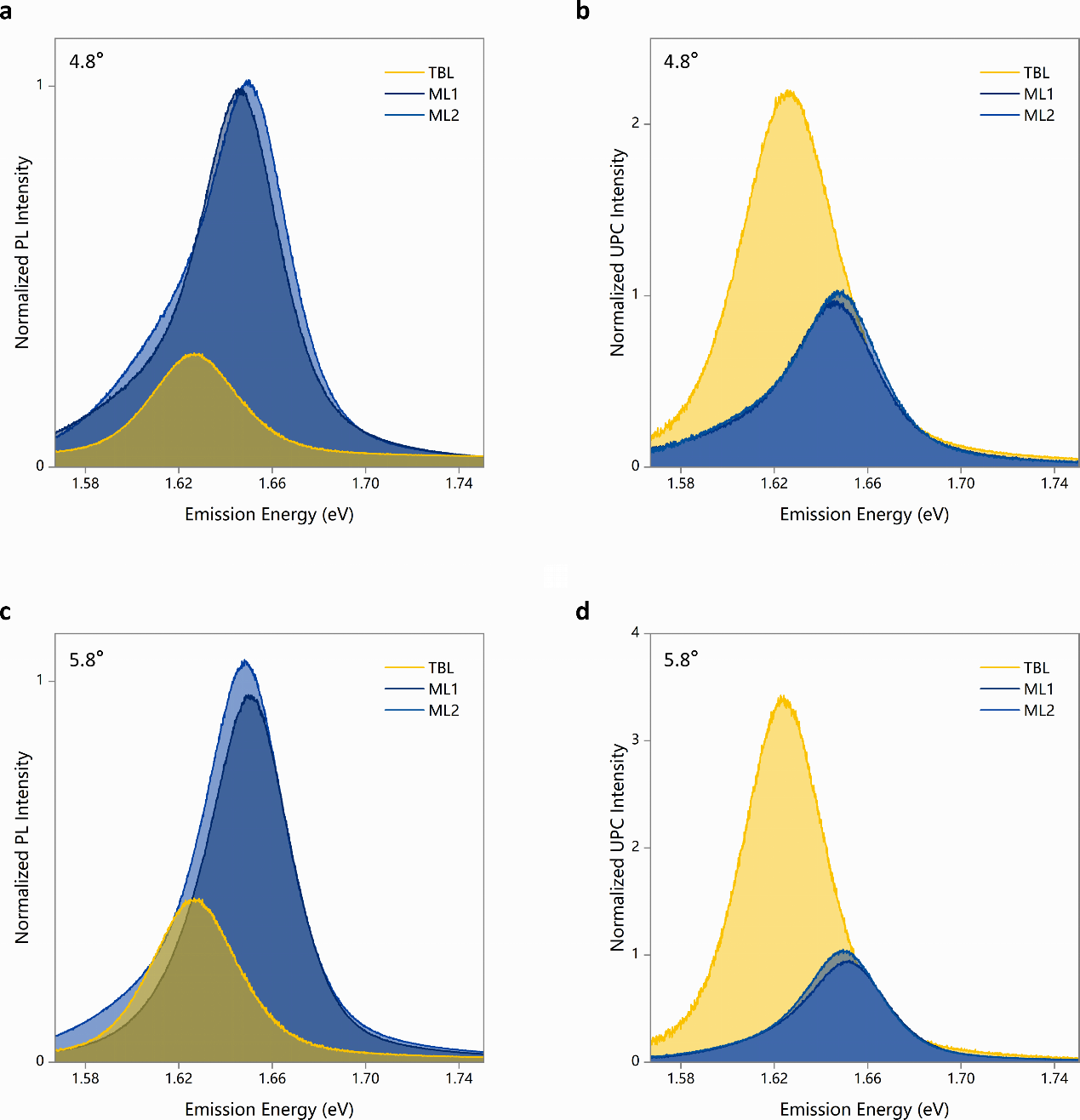


**Figure S7**: PL (**a**,**c**) and UPC (**b**,**d**) spectra of 4.8° (**a**,**b**) and 5.8° (**c**,**d**) WSe_2_ TBLs (yellow lines) and corresponding ML1 (dark blue lines), ML2 (blue lines); the average intensities in MLs are normalized as 1.

1. Summary of fitting parameters.

|  | Fitting Equations | | Parameters | | | | | |
| --- | --- | --- | --- | --- | --- | --- | --- | --- |
| Figure 2b | $I_{UPC}=\beta+\frac{\alpha-\beta}{1+exp(\frac{\Delta E-E_{0}}{dx})}$ | | α (a.u.) | β (a.u.) | E_0_ (meV) | | R^2^ | |
|  |  |  | 14.9 | 0.576 | 89.4 | | 0.9997 | |
| Figure 5a | $\frac{\partial N_{x_{0}}}{\partial t}=-AN_{x_{0}}^{4}-BN_{x_{0}}^{2}-FN_{x_{0}}+RN_{Ix}$  $\frac{\partial N_{Ix}}{\partial t}=FN_{x_{0}}-RN_{Ix}$ |  | A (cm^6^·s^-1^) | B (cm^2^·s^-1^) | F (s^-1^) | R (s^-1^) | | R^2^ |
|  |  | Top | 19×10^-22^ | 1.1 | 0 | 0 | | 0.9993 |
|  |  | Bottom | 17×10^-22^ | 1.1 | 0.81 | 0.53 | | 0.9985 |
| Figure 5b | $I_{PL}=\alpha e^{-\frac{t}{\tau_{1}}}+\beta e^{-\frac{t}{\tau_{2}}}$ |  | α (a.u.) | τ_1_ (ns) | β (a.u.) | τ_2_ (ns) | | R^2^ |
|  |  | 5.5 | 0.985 | 1.23 | 15×10^-3^ | 35 | | 0.9999 |
|  |  | 1.1 | 0.990 | 1.31 | 10×10^-3^ | 36 | | 0.9999 |
|  |  | 13.8 | 0.992 | 1.06 | 7×10^-3^ | 31 | | 0.9999 |
|  |  | ML | 0.999 | 0.85 | 1×10^-3^ | 20 | | 0.9999 |

**Table S1**: Fitting parameters in Figure 2b, 5a and 5b.

References

1. Varshni, Y. P. Temperature dependence of the energy gap in semiconductors. *Physica* **34**, 149-154 (1967).

2. Rudin, S., Reinecke, T. L. & Segall, B. Temperature-dependent exciton linewidths in semiconductors. *Physical Review B* **42**, 11218-11231 (1990).
